# Serologic Evidence of Severe Fever with Thrombocytopenia Syndrome Virus and Related Viruses in Pakistan

**DOI:** 10.3201/eid2607.190611

**Published:** 2020-07

**Authors:** Ali Zohaib, Jingyuan Zhang, Muhammad Saqib, Muhammad Ammar Athar, Muhammad Hammad Hussain, Jing Chen, Awais-ur-Rahman Sial, M. Haleem Tayyab, Murrafa Batool, Saeed Khan, Yun Luo, Cecilia Waruhiu, Zeeshan Taj, Zulfiqar Hayder, Riaz Ahmed, Abu Bakr Siddique, Xinglou Yang, Muhammad Asif Qureshi, Ikram Uddin Ujjan, Amanullah Lail, Iahtasham Khan, Sajjad-Ur-Rahman, Tao Zhang, Fei Deng, Zhengli Shi, Shu Shen

**Affiliations:** Atta-ur-Rahman School of Applied Biosciences, National University of Sciences and Technology H-12 Campus, Islamabad, Pakistan (A. Zohaib);; Wuhan Institute of Virology, Chinese Academy of Sciences, Wuhan, China (A. Zohaib, J.Y. Zhang, J. Chen, Y. Luo, C. Waruhiu, X.-L. Yang, T. Zhang, F. Deng, Z.-L. Shi, S. Shen);; University of Agriculture Faisalabad, Faisalabad, Pakistan (M. Saqib, M. Batool, M.H. Tayyab, Sajjad-Ur-Rahman);; National Institute of Virology, Dr. Panjwani Center for Molecular Medicine and Drug Research, International Center for Chemical and Biological Sciences (ICCBS) University of Karachi, Karachi, Pakistan (M.A. Athar);; Animal Health Research Center, Ministry of Agriculture and Fisheries, Muscat, Oman (M.H. Hussain);; PMAS Aird Agriculture University, Rawalpindi, Pakistan (A. Sial);; Dow University of Health Science, Karachi, Pakistan (S. Khan, M.A. Qureshi, A. Lail);; Government College University, Faisalabad, Pakistan (Z. Taj);; Quaid e Azam Medical College Bahawalpur, Bahawalpur, Pakistan (Z. Hayder);; Shifa Khana Sahib Zaman Hospital, Quetta, Pakistan (R. Ahmed); Pakistan Lab Diagnostic and Research Center, Rahim Yar Khan, Pakistan (A. Siddique);; Liaqat University of Medical and Health Science, Jamshoro, Pakistan (I.D. Ujjan);; College of Veterinary and Animal Sciences Jhang, University of Veterinary and Animal Sciences, Lahore, Pakistan (I. Khan);; National Virus Resource Center, Wuhan (F. Deng, S. Shen)

**Keywords:** Neutralizing antibodies, Pakistan, seroprevalence, severe fever with thrombocytopenia syndrome, SFTSV, SFTSV-related viruses, viruses

## Abstract

We describe the seroprevalence of severe fever with thrombocytopenia syndrome virus (SFTSV) and the association of antibody occurrence with location, sex, and age among the human population in Pakistan. Our results indicate substantial activity of SFTSV and SFTSV-related viruses in this country.

Severe fever with thrombocytopenia syndrome (SFTS) is an emerging tickborne disease caused by the SFTS virus (SFTSV; genus *Banyangvirus*, family *Phenuiviridae*, order *Bunyavirales*). The disease is prevalent in East Asia countries. It was first detected in China in 2009 and later in Japan and South Korea (*1*) and is suspected to be widely spread across other parts of the world (*2*). The recent identification of SFTSV in Xinjiang, China (*3*), expanded our awareness of epidemic areas of SFTS and suggested the possibility of SFTSV spreading to bordering countries like Pakistan. However, the presence of SFTSV in Pakistan has been unclear. We investigated the seroprevalence of SFTSV in humans in Pakistan. 

## The Study 

For this study, we randomly collected human serum samples (n = 1,657) from 4 provinces in Pakistan during 2016–2017 (Figure). All participants were farmers of livestock (sheep, goats, cattle, buffaloes, and camels). We recorded and summarized testing results by sex, age, and geographic location (Table). The collection of human serum samples and subsequent tests were reviewed and approved by the Ethics Committees of Government College University, Faisalabad, Pakistan (approval number: GCUF/MICRO/18/1598). Adult participants and parents of participants <18 years of age provided written informed consent. 

**Figure:**
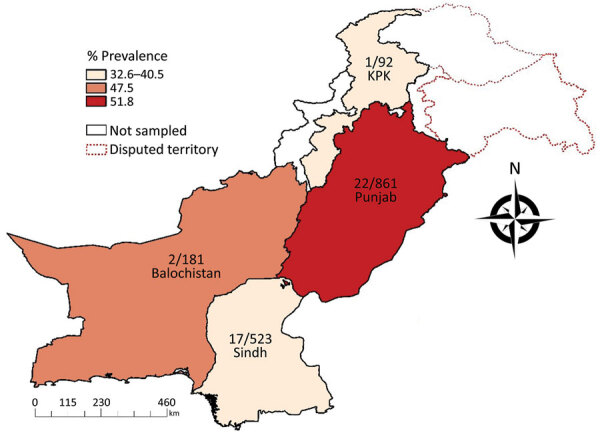
Seroprevalence of severe fever with thrombocytopenia syndrome virus in 4 provinces of Pakistan determined on the basis of ELISA detection. Numbers on map indicate microneutralization test–positive samples/total number of samples collected from the respective provinces. KPK, Khyber Pakhtunkhwa.

**Table:** Seroprevalence of severe fever with thrombocytopenia syndrome virus in Pakistan based on ELISA and MNT results

Category	ELISA				MNT		
	No. positive/no. tested	Prevalence, % (95% CI)	p value		No. positive/no. tested	Prevalence, % (95% CI)	p value
Province							
Punjab	446/861	51.8 (48.4–55.2)	<0.001		22/861	2.6 (1.6–3.8)	0.339
Balochistan	86/181	47.5 (40.1–55.1)			2/181	1.1 (0.1–3.9)	
Sindh	212/523	40.5 (36.3–44.9)			17/523	3.3 (1.9–5.2)	
KPK	30/92	32.6 (23.2–43.2)			1/92	1.1 (0–5.9)	
Sex							
M	313/733	42.7 (39.1–46.4)	0.004		17/733	2.3 (1.4–3.7)	0.619
F	461/924	49.9 (46.6–53.2)			25/924	2.7 (1.8–4)	
Age group, y							
15–24	196/413	47.5 (42.6–52.4)	0.919		8/413	1.9 (0.8–3.8)	0.120
25–34	310/669	46.3 (42.5–50.2)			21/669	3.1 (2–4.8)	
35–44	149/325	45.9 (40.3–51.4)			3/325	0.9 (0.2–2.7)	
45–54	89/184	48.4 (41–55.8)			8/184	4.4 (1.9–8.4)	
55–64	22/52	42.3 (28.7–56.8)			1/52	1.9 (0–10.3)	
>65	8/14	57.1 (28.9–82.3)			1/14	7.1 (0.2–33.9)	
Total	774/1657	46.7 (44.3–49.1)			42/1657	2.5 (1.9–3.4)	

*KPK, Khyber Pakhtunkhwa; MNT, microneutralization test.

We used a 2-step approach to detect antibodies against SFTSV. First, we screened the samples for SFTSV IgG by using a SFTSV human commercial ELISA kit (NZK Bio-tech, https://hbnzk.com), which employs SFTSV nucleocapsid protein (NP) as the viral antigen. To set up negative and positive controls, we used serum samples from 3 healthy persons from Wuhan, China (*4*), and serum samples from 2 convalescent SFTS patients from Wuhan archived in the National Virus Resource Center (accession nos. YB17WIVS286, YB17WIVS294). Following manufacturer instructions (Appendix), we tested serum samples at 1:20 dilution; we considered samples IgG-positive when the absorbance was >2.1 times the mean absorbance of the negative control. Samples with optical density values >0.41 were considered SFTSV IgG–positive (Appendix Figure 1). We used an immunofluorescence assay modified from a previous study (*5*) to verify the validity of the commercial ELISA. 

In the second step, we used a microneutralization test (MNT) assay to distinguish SFTSV-specific neutralizing antibodies from SFTSV-related viruses, as described elsewhere (*6*); we estimated SFTSV prevalence from ELISA and MNT results within 95% CIs. We performed statistical analysis of the data using the χ^2^ test or Fisher exact test to explore the association of SFTSV with age, sex, and location. We performed the analysis in R version 3.5.1 with the Epicalc package version 2.15.1.0 (http://www.r-project.org). 

The ELISA revealed a high seroprevalence (46.7%, 95% CI 44.3%–49.1%) of SFTSV in Pakistan (Table; Figure). Spatial distribution analysis indicated the highest prevalence (51.8%, 95% CI 48.4%–55.2%) in the Punjab province, followed by Balochistan (47.5%, 95% CI 40.1%–55.1%), Sindh (40.5%, 95% CI 36.3%–44.9%), and Khyber Pakhtunkhwa (32.6%, 95% CI 23.2%–43.2%). The prevalence was significantly higher (p = 0.004) in women (49. 9%, 95% CI 46.6%–53.2%) than in men (42.7%, 95% CI 39.1%–46.4%). The seroprevalence increased with age, but not uniformly; the highest seroprevalence (57.1%, 95% CI 28.9%–82.3%) was recorded in samples from persons >65 years of age. A technician unaware of the ELISA results and research details performed a single-blind test with 90 ELISA-negative and 252 ELISA-positive samples, randomly selected. This test confirmed results for 100% of the ELISA-negative samples and 212 (84.1%) of 252 of the ELISA-positive samples (Appendix Figure 2). 

We confirmed SFTSV infection using MNT, which revealed a low prevalence (2.5%, 95% CI 1.9%–3.4%) in Pakistan (Table; Appendix Figure 3). Women had a higher occurrence of anti-SFTSV neutralizing antibodies (2.7%, 95% CI 1.8%–4.0%) than men (2.3%, 95% CI 1.4%–3.7%), however, this difference was not significant (p = 0.619). Neutralizing antibodies were detected in all age groups. Furthermore, we performed MNT for a novel virus, Guertu virus (GTV) (*4*), which is closely related to SFTSV, on 10 randomly selected serum samples that tested positive for SFTSV neutralization and 10 SFTSV IgG–positive samples that were negative for neutralization (Appendix Figure 3). All 10 samples that tested negative on the SFTSV MNT also tested negative on the GTV MNT. However, 3 of the 10 samples that tested positive on the SFTSV MNT also exhibited neutralization to GTV; the other 7 samples tested negative for neutralizing GTV. 

## Conclusions 

This study highlights the activity of SFTSV and its substantial risks to the population in Pakistan. The observed high ELISA-based prevalence could be ascribed to the study population in this survey being livestock farmers, who could be more frequently exposed to tick vectors and livestock reservoirs. Higher estimates of SFTS prevalence in the Punjab province of Pakistan could be attributed to the high proximity of human and livestock populations in this region. Higher prevalence among women than among men was expected because livestock is mostly tended by female farmers. 

In ELISA-based estimates of SFTSV in the human population reported from different areas of East Asian countries, seroprevalence has ranged from 0.23% to 9.17% in China (*7*), from 1.9% to 7.7% in Korea (*8*, *9*), and from 0.14% to 0.3% in Japan (*10*, *11*). In contrast to the findings from these reports (*7**–**11*), our study found a markedly high ELISA-based prevalence of SFTSV (46.7%) in Pakistan. The use of different SFTSV antibody detection methods may have led to the observed differences in results (*11*); nevertheless, results of the blind test using an immunofluorescence assay still suggested a high prevalence, such that 84.12% of the randomly selected ELISA-positive serum samples could react with the SFTSV antigen. Therefore, the low prevalence of neutralizing antibodies (2.5%) against SFTSV suggests the possibility of cocirculating antigenicity-related viruses that were not discernable in the indirect ELISA tests. 

The genus *Banyangvirus* currently includes the Bhanja and SFTS/Heartland groups. The 5 viruses of the SFTS/Heartland group have a wide geographic distribution. SFTSV is found mostly in China, Japan, and South Korea (*1*); GTV in northwestern China (XJUAR) (*4*), Heartland virus in the United States (*12*); Hunter Island group virus in Australia (*13*); and Malsoor virus in India (*14*). 

Similar to findings in our study, a high seroprevalence (19.8%) of GTV was detected among the local residents of Guertu County in Xinjiang, China; however, only 3 (0.65%) of the 465 serum samples had neutralizing antibodies against GTV (*4*). Antigenic cross-reactivity between SFTSV and GTV was suspected because cross-neutralization was observed in mouse serum (*4*). However, serologic investigation of other bunyaviruses is limited, and serotypes of the 2 viruses, as well as other related viruses in the SFTS/Heartland group, remain unclear. 

Our subsequent study found that a few serum samples exhibiting neutralization to SFTSV also exhibited cross-neutralization to GTV. All of these results indicate the presence in Pakistan of SFTSV and SFTSV-related viruses that might share antigenic similarity and could induce antibodies exhibiting cross-reactivity with each other. In addition, a recent study reported suspected clinical SFTS cases in Pakistan; however, they were not confirmed using serologic or molecular tests (*15*). 

Our findings suggest the potential risk for infection from SFTSV and SFTSV-related viruses in Pakistan. Further work on the discovery, identification, and ecology of these viruses in ticks, animal hosts, and human patients is needed because the viruses pose potential threats to public health. 

Appendix Additional information for study of serologic evidence of severe fever with thrombocytopenia syndrome virus and related viruses in Pakistan.
