# Supplementary material for: Serologic Evidence of Severe Fever with Thrombocytopenia Syndrome Virus and Related Viruses in Pakistan
Source: Emerg Infect Dis. 2020 Jul;26(7):1513–6. doi: 10.3201/eid2607.190611 (PMC7323538; doi:10.3201/eid2607.190611)
Supplement: Appendix — Additional information for study of serologic evidence of severe fever with thrombocytopenia syndrome virus and related viruses in Pakistan. [file 19-0611-Techapp-s1.pdf]

Article DOI: https://doi.org/10.3201/eid2607.190611

Serologic Evidence of Severe Fever with Thrombocytopenia Syndrome Virus and Related Viruses in Pakistan

Appendix

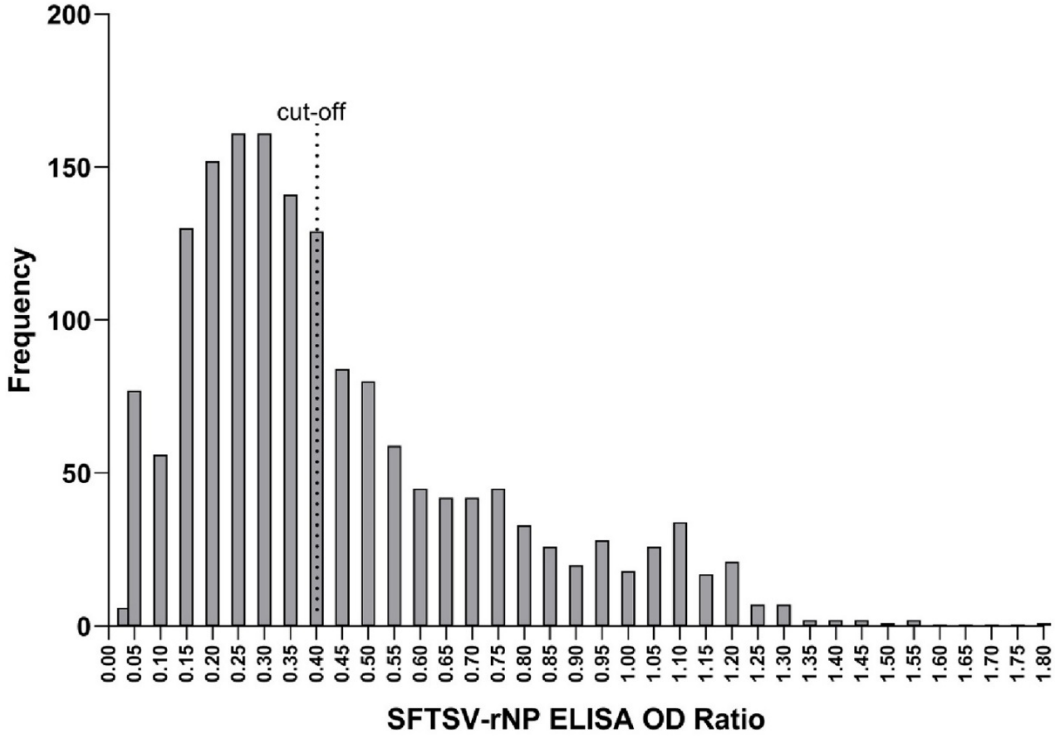

Appendix Figure 1. A plot showing the frequency of optical density (OD) values from all 1657 human serum samples. The cutoff value at which samples were considered anti-SFTSV IgG-positive was set at an OD value ≥0.41, according to the ELISA kit instructions.

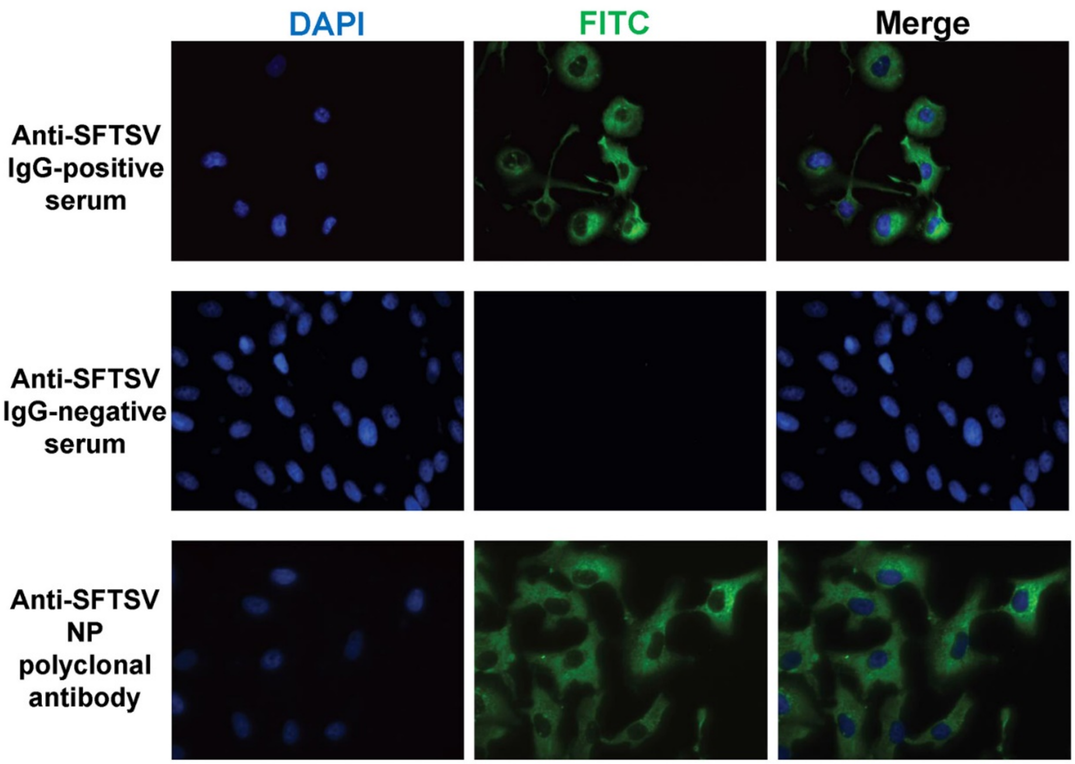

Appendix Figure 2. Images of indirect immunofluorescence assay (IFA) that was used to verify the results from ELISA. Representative images are shown, including an ELISA-positive serum sample that was also detected anti-SFTSV IgG-positive by IFA (upper panel); an ELISA-negative serum sample which was detected anti-SFTSV IgG-negative (middle panel); and an anti-SFTSV nucleoprotein polyclonal antibody (*4*) used as a positive control (bottom panel).

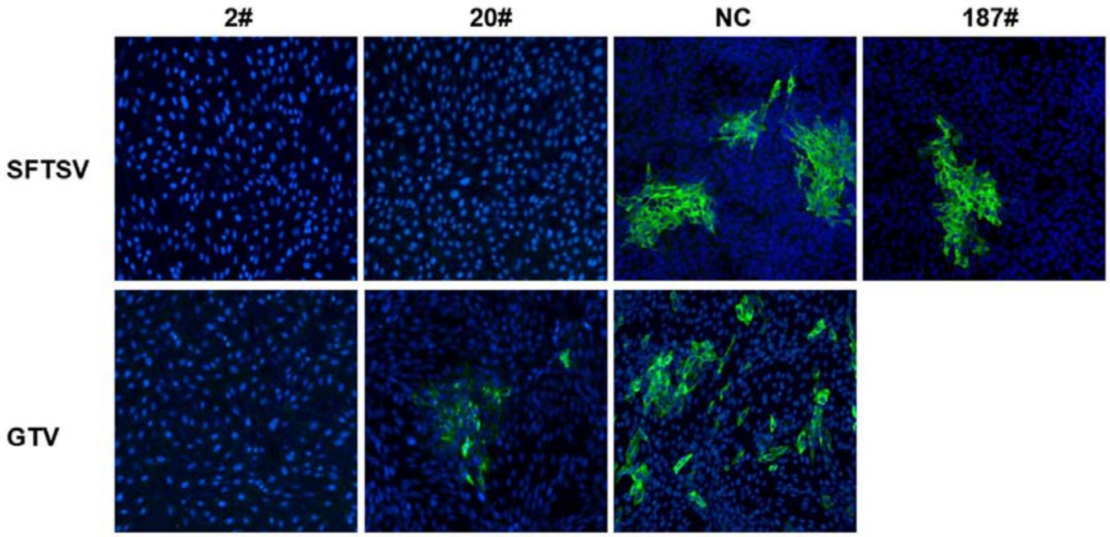

Appendix Figure 3. Images of microneutralization (MNT) assay showing the representative results of SFTSV or GTV infection in cells after incubating with human serum samples. Neither SFTSV nor GTV infection could be observed if serum sample could neutralize both viruses. Of the 10 serum samples MNT-positive to SFTSV, 3 samples were detected having cross-neutralization to GTV. Images from sample 2# were shown. The other 7 serum samples could neutralize SFTSV but could not react with GTV. Images from sample 20# show that SFTSV infection was prevented and GTV infection was observed. SFTSV and GTV infection were both observed in the negative control (NC) when viruses (100 $TCID_{50}$) were mock incubated. SFTSV infection could be observed in cells when viruses were incubated with anti-SFTSV IgG-positive samples, but they had no neutralization to the virus (187#).

Manufacturer Instructions

The following pages show the manufacturer instructions for the ELISA used in this study.

新纵科
NZK biotech

NovaStar®

Cat. NZK-E17003

For Research Use Only

NZK biotech

Severe fever with thrombocytopenia syndrome virus (SFTSV) Human IgG ELISA kit

Product Manual

I. Intended Use

The Severe Fever with Thrombocytopenia Syndrome Virus (SFTSV) Human IgG ELISA Kit is an in vitro enzyme immunoassay for the specific determinations of human anti-SFTSV IgG in serum.

II. Principle

The Severe Fever with Thrombocytopenia Syndrome Virus (SFTSV) Human IgG ELISA Kit is a solid phase assay based on an indirect method that utilizes purified recombinant SFTSV nucleoprotein (NP) to detect antibodies in human serum samples. The protein is coated on the microtiter plate to create the solid phase. Non-specific binding is blocked by a blocking buffer. Serum samples are diluted with dilution buffer and incubated in wells. Then, the wells are washed, and incubated with the second antibody, anti-human IgG antibody conjugated with horseradish peroxidase (HRP). During the incubation, anti-SFTSV NP antibodies in human serum were captured, then the specific IgG was recognized with the second antibody. Reaction between HRP and substrates results in color development with intensities proportional to the amount of anti-SFTSV NP antibody present in serum samples. The results can be measured by the absorbance with a microplate reader. Serum samples positive for anti-SFTSV NP IgG can be determined by comparing the absorbances with those of the negative control.

III. Reagents and Material

Each kit includes reagents sufficient for 96 wells. The expiration date for the complete kit is stated on the outer box label and the recommended storage temperature is 2-8 °C.

A. Materials Provided

(1) SFTSV NP coated microtiter plate, 1 plate (96 wells: 8 wells × 12 strips)

(2) HRP-conjugated anti-human IgG antibody, 12 mL

(3) Sample dilution buffer, 12 mL

(4) Washing buffer (20×), 50 mL

(5) Substrate solutions

Solution A, 5 mL

Solution B, 5 mL

(6) Stop buffer, 5 mL

(7) One zip-lock bag

B. Equipment and Materials Required but not Provided

(1) Pipette, micropipette, and tips

(2) Microplate reader

(3) Incubator (37±1°C)

(4) Absorbent paper

IV. Precautions

- Do not mix reagents from different kit lots.

- Do not use reagents beyond expiration date on label.

- In order to avoid reagent contamination, use disposable pipette tips and/or pipettes.

- Do not expose Substate Solution to strong light during storage or incubation.

- Avoid contact of Substrate and Stop Solution with skin or mucous membranes. If these reagents come into contact with skin, wash thoroughly with water. Do not pipette by mouth. Do not smoke, eat, or drink in area where specimens or kit reagents are handled. All blood fluids should be considered as potentially infectious.

- Avoid contact of Substrate Solution and Stop Solution with any metal surface. Disposable glassware or test tubes are recommended for handling the Substrate Solution. If non-disposable glassware is used, it must be acid

washed and thoroughly rinsed with distilled, deionized water.

- Do not use the Substrates if its color is changed to thick blue.

V. Specimen Collection and Handling

This kit is only for use of detection of anti-SFTSV IgG antibody in human serum. Remove the serum from the clot or red cells, respectively, soon after clotting and separation. Samples containing a visible precipitate must be clarified prior to use in the assay, and should not be contaminated with microorganisms. Fresh samples could be stored at 2-8°C for one week. If the length of time between sample collection and assay is to exceed 24 hours, samples are suggested to be stored frozen under-20℃ or lower temperature. Excessive freeze-thaw cycles should be avoided. Do not use grossly hemolyzed or lipemic specimens. Prior to assay, frozen samples should be brought to room temperature slowly, and gently mixed by hand. Do not thaw samples in a hot bath. Do not vortex or sharply agitate.

VI. Procedure

Notes: The microtiter plates should be brought to room temperature 30 min before use. For thorough mixing, the microtiter plate can be gently agitated on a plate mixer or by mixing the plate sporadically by hand. Dilute the Washing Buffer (20×) in ddH_2O to working concentration (1×), which should be prepared directly before the assay.

A. The serum samples of positive control and negative control, and blank should be set up. Pipette 100μL of each into one well. Samples must be diluted in the Dilution Buffer (v/v=1:1 for the first test), 100μL per well.

B. Mix, seal the microtiter plate in the zip-lock bag, and then incubate for 30min at 37°C.

C. Remove sample solution and control and wash the wells 6 times with

200 μL of Washing Buffer (1×). Let the plate stand for 15-30sec for each time. Between the separate washing steps, empty out the microtiter plate and vigorously tap onto paper towel, especially after the last washing.

D. Pipette 100 μl of HRP labelled anti-human IgG antibody into one well. Mix, seal the microtiter plate in bag and incubate for 30 min at 37℃.

E. Remove the solution and wash the wells 6 times as described above (**C**) (It is especially important after this step to thoroughly empty out the remaining fluid before adding the substrate).

F. Add 50 μl of Solution A and 50 μl of Solution B into each well. Seal in bag and incubate at 37℃for 6-8 min.

G. Add 100 μl of Stop Solution into each well in the same order as for substrate. Tap plate gently to mix.

H. Measure the absorbance at 450 nm with a microplate reader. The absorbance should be read within 15 minutes after the completion of the assay. It may be read up to 1 hour after addition of Stop Solution if wells are protected from light at room temperature.

VII. Results

Record the absorbance at 450 nm for each sample well.

Cut-off value equals to 2.1 folds by the mean absorbance of negative controls (N) (Cut-off value =2.1×N). If mean value (N) was less than 0.05, the cut-off value equals to 2.1 by 0.05 (0.105).

The absorbance of Negative control should be no more than 0.2, or else the assay must be repeated. Samples are considered negative if absorbance < Cut-off value, and are considered positive if absorbance ≥ Cut-off value.

VIII. Contact Us

URL: https://hbnzk.com/products/materials

Hubei Xinzongke Viral Disease Control Bio-Tech Ltd.

B21, High-tech equipment park,

818, Gaoxin Avenue, East lake new technology development zone, Wuhan

Hubei, China

Tel: +86 27 87201009
